# A NIR light activated self‐reporting carbon dots assembly as phototheranostics for tumor photodynamic therapy

**DOI:** 10.1002/smo.20240049

**Published:** 2024-10-21

**Authors:** Ziyu Zhao, Tiejin Chen, Jian Li, Xiaokuang Xue, Jiechao Ge, Pengfei Wang

**Affiliations:** ^1^ Key Laboratory of Photochemical Conversion and Optoelectronic Materials Technical Institute of Physics and Chemistry Chinese Academy of Sciences Beijing China; ^2^ School of Future Technology University of Chinese Academy of Sciences Beijing China

**Keywords:** carbon dots, light response, photodynamic therapy, self‐report

## Abstract

Photodynamic therapy (PDT) has emerged as a promising protocol for cancer therapy. However, real‐time monitoring of PDT progress and accurate determination of the optimal treatment timing remain challenges. In this work, we selected carbon dots (CDs) and new indocyanine green (IR820) as building units to fabricate a smart nanotheranostics (CDs‐IR820 assembly) with the characteristics of controlled release and real‐time imaging to solve the time gap between diagnosis and treatment. The fabricated CDs‐IR820 assembly locked the photosensitivity of the CDs and could degrade under 750 nm laser irradiation to achieve controlled release of the CDs, thus used for cell imaging and producing single oxygen under the white light. Besides, the released CDs could migrate from the mitochondria to the nucleus during the PDT process, indicating the cell activity, which facilitated the regulation of treatment parameters to achieve the precise PDT for cancer.

## Graphical abstract

We develop a smart nanotheranostics (CDs‐IR820 assembly) with the characteristics of controlled release and real‐time imaging. Upon 750 nm laser irradiation, the inactive CDs in assembly could be triggered, thereby not only producing ^1^O_2_ to eliminate tumor cells under white light, but also functioning as a self‐reporter to monitor the PDT process.

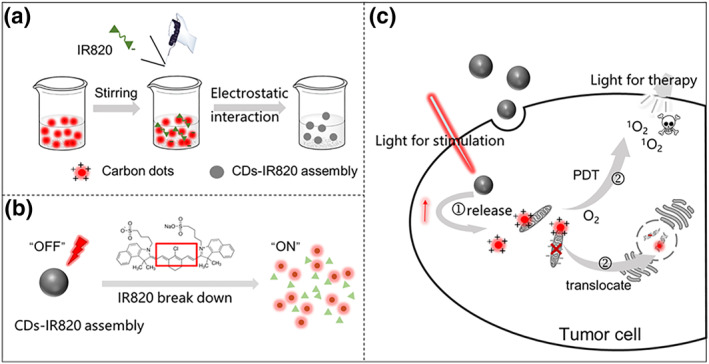

## 1 INTRODUCTION

Cancer remains one of the foremost challenges to human health, accounting for millions of fatalities and incurring a staggering economic burden of trillion dollars annually.^[1]^ Photodynamic therapy (PDT), as a noninvasive protocol, has been becoming one of the most promising therapeutic strategies alongside surgery, radiotherapy and chemotherapy.^[2]^ PDT offers numerous advantages that have attracted more and more attentions, such as high spatiotemporal selectivity, minimal side effects for the normal tissue and great potential for synergistic or repeat therapy.^[3]^ In PDT, photosensitizers (PSs) are activated by a specific wavelength light and subsequently react with the surrounding oxygen to generate reactive oxygen species (ROS), which serve as the agents that kill tumors.^[4]^ Furthermore, the remarkable optical properties of PSs enable them to function as luminous agents for fluorescence imaging.^[5]^ Therefore, PSs exhibit significant potential for theranostic applications owing to the aforementioned intrinsic properties. Specifically, PSs have the capacity to achieve fluorescence imaging guided PDT.^[6]^ However, there is usually a time gap between the fluorescence imaging and PDT that causes vague guidance during the therapeutic process, particularly at the outset or conclusion of the therapy.^[7]^ Completing the therapy too early may result in inadequate PDT, while concluding it too late may expose normal tissues to excessive high‐energy light, thereby compromising the efficacy of PDT.

To overcome this issue, substantial efforts have been invested in the design and development of PSs, particularly the self‐reporting PSs.^[8]^ In 2019, Tang's group reported a novel class of cationic aggregation induced emission organic molecules (AIEgens) with the capability of self‐reporting to monitor the cell apoptosis process triggered by PDT in situ, paving a new way for the visualization of PDT, thereby enabling the precise control of the phototoxicity dose.^[9]^ Recently, Peng and his colleagues constructed a nucleic acid‐based fluorescent probe, SPP, capable of cascade migration from the mitochondrion to the nucleus upon exposure to light. Through the integration of a PS into the SPP, the resultant PS (MTPA‐SPP) can generate ROS in mitochondria under light irradiation, followed by migration to the nucleus, thereby augmenting the efficiency of cancer therapy.^[10]^ Although the self‐reporting PSs, particularly the organic small molecule PSs, have achieved significant advancements, there is still a considerable scope to design and fabricate the novel nanomaterials as self‐reporting PSs for precise tumor PDT.

Carbon dots (CDs), as a new member of carbon nanomaterials, have garnered significant attention over the past decades because of their unique physical structures and excellent optical properties.^[11]^ Furthermore, CDs have been widely applied in various fields, such as sensors,^[12]^ anti‐bacteria agents,^[13]^ biomedicine and so forth.^[14]^ In 2014, our group reported a red emissive CDs PSs with positive surface charge and high quantum yield of singlet oxygen.^[15]^ Since then, substantial attention has been focused on CDs as a novel PSs for tumor PDT.^[16]^ Compared to traditional PSs, CDs are easily fabricated and exhibit tunable photoluminescence behaviors, great biocompatibility, high photostability and remarkable solubility.^[17]^ In addition, CDs as bioimaging agents have been widely used for treatment monitoring.^[18]^ For example, Li's group developed a kind of fluorescent CDs capable of shutting between mitochondria and the nucleolus utilized for in situ visualization of cell viability.^[19]^ These characteristics of CDs are especially compelling for the design of phototheranostics with real‐time imaging in situ for PDT. However, highly efficient phototheranostics based on CDs enabling sufficient PDT and self‐reporting the therapeutic process in situ are still highly desired.

**SCHEME 1:**
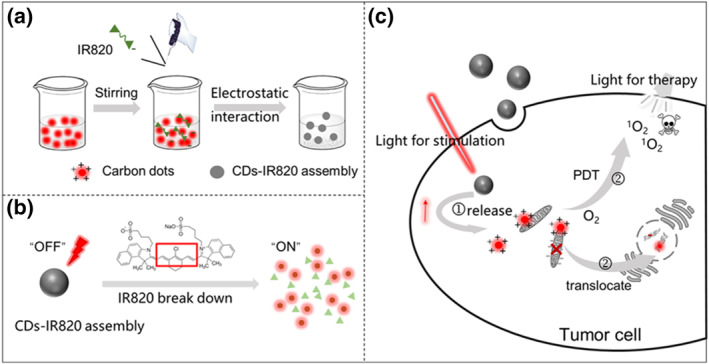
(a) The method of fabrication of CDs‐IR820 assembly; (b) the diagram of the mechanism of controlled release; (c) the schematic illustration of CDs‐IR820 assembly for controlled release, real time imaging and PDT of cancer.

Inspired by the Tang group's work, we presupposed that the red emissive CDs may have the potential to real‐time monitor the cell apoptosis process induced by PDT. To prove our assumptions and simultaneously further enhance the biocompatibility and minimize the side effect of phototoxicity of the CDs, we selected new indocyanine green (IR820, an analog of FDA‐approved indocyanine green) and the CDs served as the block unit to fabricate the self‐assembly CDs‐IR820 via electrostatic interaction effect (Scheme 1a). Notably, the CDs were quenched by IR820 in the assembly due to the Förster Resonance Energy Transfer (FRET) interaction which simultaneously locked the photosensitivity of the CDs. Under the irradiation of 750 nm laser, IR820 was broken down, releasing the CDs (Scheme 1b). The released CDs with recovered photosensitivity could kill the tumor cells by the produced ^1^O_2_ under light illumination. Interestingly, the CDs could migrate from mitochondria to the nucleus during the PDT process, reporting the death of tumor cells (Scheme 1c). Therefore, the CDs‐IR820 assembly could realize the real‐time self‐reporting of the PDT process through monitoring the translocation of the CDs, providing a new use of CDs for designing precise PDT with high security.

## 2 RESULTS AND DISCUSSION

### 2.1 Synthesis and characterization of the CDs‐IR820 assembly

**FIGURE 1:**
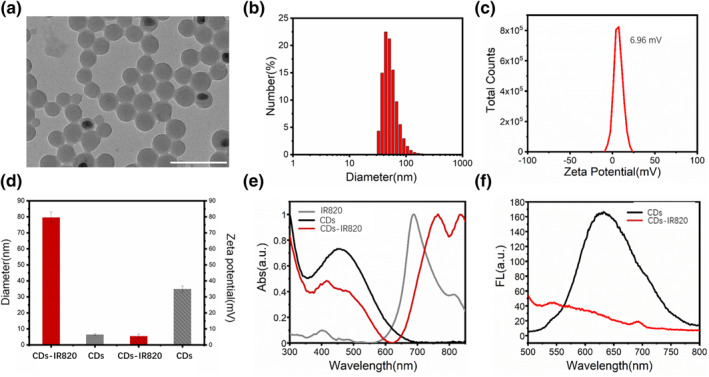
(a) Transmission electron microscopy (TEM) image of CDs‐IR820 assembly; (b) The dynamic light scatter (DLS) analysis and (c) the Zeta potential of CDs‐IR820 assembly; (d) The comparison between CDs‐IR820 assembly and carbon dots (CDs) in diameter and Zeta potential; (e) Absorption spectra of individual IR820, CDs and CDs‐IR820 assembly; (f) Fluorescence spectra of individual CDs and CDs‐IR820 assembly. Scar bar: 200 nm.

IR820 was conjugated with CDs to fabricate the CDs‐IR820 assembly through electrostatic interaction. The transmission electron microscopy (TEM) image showed that the CDs‐IR820 assembly had excellent dispersion and uniform spherical shape with a mean diameter of 64.8 nm (Figure 1a and Figure S1). As indicated by the dynamic light scatter analysis (Figure 1b), the hydrodynamic diameter of the CDs‐IR820 assembly was 80 ± 3 nm, which was consistent with the results of TEM. Furthermore, the fabricated CDs‐IR820 assembly exhibited a near neutral surface potential of approximately 6.96 mV (Figure 1c). Compared to pure CDs, there was a noticeable increase in size coupled with a transition from a strong positive charge to a near neutral charge (Figure 1d). The absorbance spectrum of the fabricated CDs‐IR820 featured the characteristic absorption peak of CDs (around at 460 nm) and IR820 (approximately at 750–850 nm), illustrating the successful synthesis of the CDs‐IR820 assembly (Figure 1e). It was worth noting that there was a red shift (approximately 50 nm) in the characteristic absorption peak of IR820 after assembly, attributed to the occurrence of J‐type aggregation of aromatic structure in the IR820 during assembly process.^[20]^ The emission spectrum of CDs‐IR820 assembly revealed that the fluorescence of CDs was quenched after conjugating with IR820 because of the FRET process,^[21]^ signifying a transition of luminescence from “ON” state to “OFF” state due to the successful fabrication of the CDs‐IR820 assembly (Figure 1f).

**FIGURE 2:**
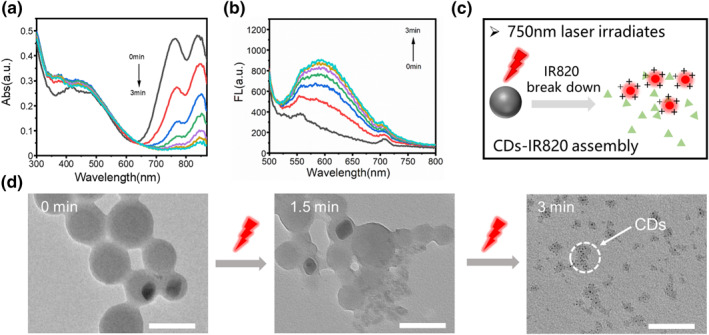
(a) The absorption spectra of CDs‐IR820 assembly under 750 nm laser irradiation at different time (0–3 min); (b) The emission spectra of CDs‐IR820 assembly under 750 nm laser irradiation at different time; (c) The diagram of the controlled release of carbon dots (CDs) under 750 nm laser irradiation; (d) The transmission electron microscopy (TEM) image of CDs‐IR820 assembly under 750 nm laser irradiation at different time. Scar bar: 100 nm.

The capability of light‐responsive release of CDs from the CDs‐IR820 assembly was further investigated. Upon 750 nm (100 mW/cm^2^) laser irradiation, as shown in Figure 2a, the characteristic absorption peak at around 750–850 nm gradually diminished with the increasing irradiation time to 3 min, indicating the decomposition of IR820 owing to the rupture of the double bond within the IR820 molecule. Simultaneously, the fluorescence intensity of the CDs gradually increased (approximately 4 times to the original level), illustrating the release of CDs from the CDs‐IR820 assembly (Figure 2b). Consequently, a transition of the luminescence state of CDs from “off” to “on” was realized due to the breakdown of IR820 under the laser irradiation (Figure 2c). The TEM images further verified the light‐controlled release process of the CDs‐IR820 assembly. As depicted in Figure 2d, the integrity of the CDs‐IR820 assembly was destroyed and the CDs were partially released after 1.5 min of irradiation. Upon 3 min of irradiation, free CDs could be clearly observed, indicating the complete dissembly of CDs‐IR820 assembly.

The electron spin resonance (ESR) technique was employed to detect the ^1^O_2_ generated by the CDs‐IR820 assembly under irradiation. 2,2,6,6‐Tetramethylpiperidine was used as the ^1^O_2_ trapper, respectively. As shown in Figure S1, there was no obvious change in the ESR spectra under the 750 nm laser irradiation. However, the characteristic ^1^O_2_‐induced signal was observed in the ESR spectra under the 750 nm laser followed by xenon lamp irradiation, and its intensity increased with the increase in the irradiation time. Furthermore, the ABDA was used as a specific chemical trapping agent of ^1^O_2_ to examine the capacity of the CDs‐IR820 assembly for generating the ^1^O_2_. As shown in Figure S2, under the 750 nm laser followed with the xenon lamp irradiation, the absorbance of the ABDA solution at 378 nm decreased gradually with the prolonged irradiation time, indicating the degradation of ABDA by ^1^O_2_ generated from the CDs‐IR820 assembly. However, negligible decrease could be observed under only 750 nm laser irradiation, which was consistent with the results of ESR. These findings collectively demonstrated that the 750 nm laser only served as the stimulating light to control the release of CDs. Subsequently, the released CDs were capable of generating a substantial quantity of ^1^O_2_ under the xenon lamp irradiation. This process indicated that the initiation point of the PDT could be precisely selected by altering the light source with minimal superfluous photo damage.

### 2.2 Photo‐controlled release of CDs in cells

**FIGURE 3:**
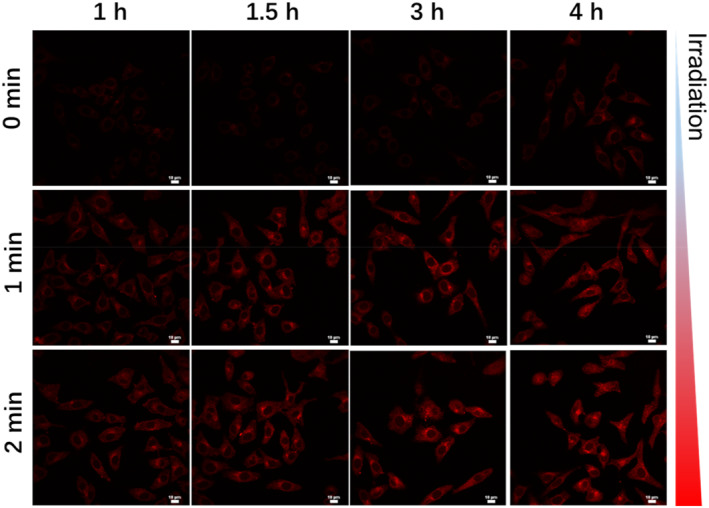
Confocal microscopy images of HeLa cells incubated with CDs‐IR820 assembly at different periods of incubation time from 1 to 4 h, and at different periods of irradiation time from 0 to 2 min. Scale bar: 10 μm.

Investigation of the controlled release process of the CDs‐IR820 assembly was conducted at the cellular level. The CDs‐IR820 assembly was co‐incubated with HeLa cells for 1 h, 1.5 h, 3 h and 4 h, respectively, and subsequently imaged using confocal microscopy. As shown in Figure 3, no detectable red fluorescence of the CDs could be observed before the 750 nm laser irradiation. As anticipated, the increased red fluorescence of the CDs could be detected with the increasing prolonged irradiation time from 0 to 2 min, indicating that the disassembly of the CDs‐IR820 assembly could be achieved under the 750 nm laser irradiation within the cells. Remarkably, the red fluorescence was observed in the center of certain cells with the prolonged observation time, which was potentially associated with the cellular activity induced by the photodynamic effects under the confocal laser excitation (Figure S3).

### 2.3 PDT in in vitro

The capacity of CDs‐IR820 to generate ROS within the cells was initially evaluated by the 2′,7′‐dichlorodihydro‐fluorescein diacetate (DCFH‐DA) staining. As shown in Figure S4, no green fluorescence signal was collected under the 750 nm laser irradiation within 3 min. As anticipated, upon the 750 nm laser followed with the xenon lamp irradiation, the green fluorescence signal emerged within the cells, and the increased intensity with the prolonging irradiation time could be detected, thus demonstrating that the CDs were released under 750 nm laser irradiation and could generate abundant ROS upon the xenon lamp irradiation.

**FIGURE 4:**
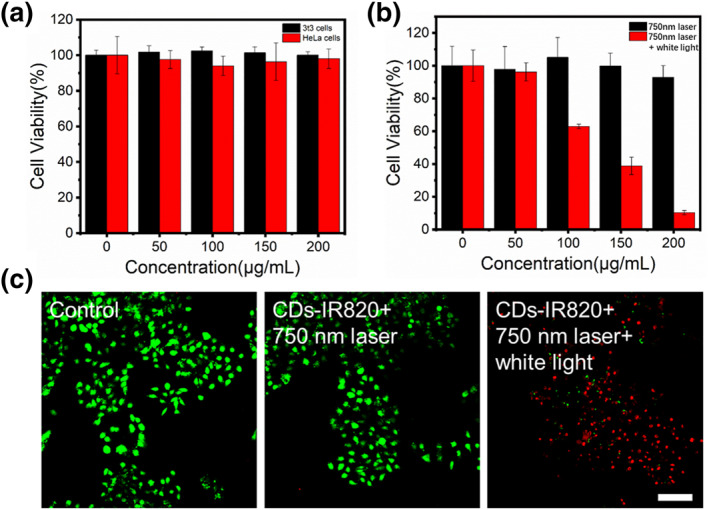
(a) Relative viabilities of HeLa and 3t3 cells incubated with different concentrations of CDs‐IR820 assembly; (b) Relative viabilities of HeLa cells incubated with different concentrations of CDs‐IR820 assembly post various treatments; (c) FL images of Calcein AM/PI co‐staining HeLa cells. Scar bar: 100 μm.

Subsequently, the cytotoxicity and antitumor effect of CDs‐IR820 assembly for PDT in vitro were evaluated through MTT assays. As shown in Figure 4a, after CDs‐IR820 assembly and tumor cells co‐cultured 24 h without irradiation, 95% of cell viability could be observed, illustrating the negligible dark cytotoxicity and good biocompatibility of CDs‐IR820 assembly. Furthermore, there was no discernible decrease in cell viability under the 750 nm laser irradiation, even the concentration of the CDs‐IR820 reached 200 μg/mL. In contrast, HeLa cells exhibited a significant concentration‐dependent cell inhibition effect on the 750 nm laser followed by xenon lamp irradiation. At concentrations of 200 μg/mL, the cell survival rate was less than 15%, suggesting that the CDs‐IR820 assembly could achieve an excellent PDT effect (Figure 4b). To further assess the anticancer efficacy of the CDs‐IR820 assembly, HeLa cells incubated with CDs‐IR820 assembly were treated with light and then stained with calcein‐AM/PI. As shown in Figure 4c and Figure S6, only green fluorescence was observed in the control, light‐only, CDs‐IR820 assembly‐only or CDs‐IR820 + 750 nm laser groups. However, a homogeneous red fluorescence could be observed in the CDs‐IR820 with 750 nm laser and xenon lamp irradiation, demonstrating the complete killing of tumor cells due to the ROS production of the CDs‐IR820 assembly with irradiation.

### 2.4 Self‐reporting the cell death

**FIGURE 5:**
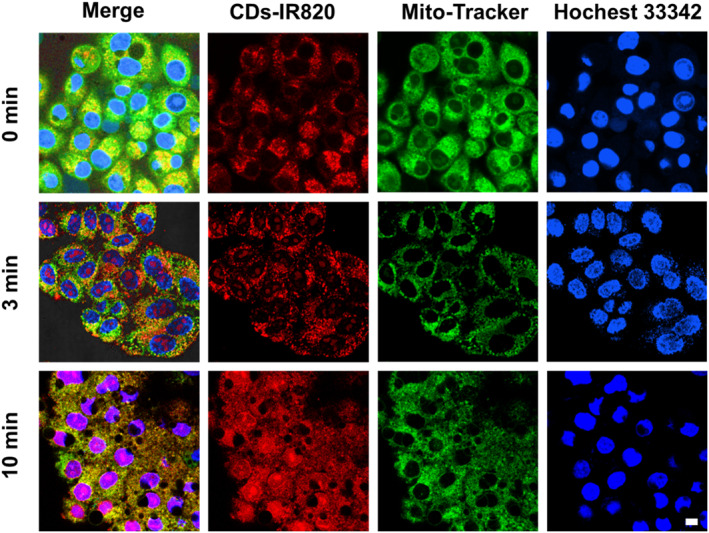
Confocal images of HeLa cells under different xenon lamp irradiation times stained with CDs‐IR820 (red), Mito‐Tracker (green) and Hochest 33342 (blue). Scar bar: 10 μm.

The fantastic cell uptake imaging and the excellent PDT activity provided the CDs‐IR820 assembly a foundation for serving as a smart nanotheranostics with the capability of monitoring the cell death during the PDT process. Aiming to obtain the real‐time therapeutic feedback for optimizing the treatment schedule that could avoid overtreatment and minimize potential side effects, the co‐localization experiments of the CDs‐IR820 assembly were conducted with the commercial fluorescent dyes, Mito‐Tracker green and Hoechst 33342, as the mitochondria and nucleus indicators. As shown in Figure 5, the red fluorescence of the CDs overlapped well with the green fluorescence of the mitochondria indicator, indicating that the CDs were mainly located in the mitochondria initially. However, after 3 min of white light illumination, the red fluorescence was observed to partly overlap with the blue fluorescence of the nucleus indicator, indicating that some CDs migrated into the nucleus from mitochondria. Moreover, with the prolonged illumination to 10 min, the blue fluorescence was entirely covered by the red fluorescence, indicating that more CDs transferred into the nucleus and filled the entire nucleus. Meanwhile, the morphology of the nucleus was significantly changed, indicating that cell death occurred. These findings demonstrated that the CDs‐IR820 assembly could self‐report the cell activity during the PDT process by migrating the CDs from mitochondria to the nucleus. Compared to the previous studies (Table S1), the majority of research pertinent to self‐reporting is based on the organic small molecules, including the AIE molecules and metal complexes. Unfortunately, the relatively complex synthesis method and poor biocompatibility hinder their potential for further clinical application. Additionally, some researchers have investigated the inorganic materials such as black phosphorus and gold nanoparticles. However, all these studies achieved the self‐reporting by loading the fluorescent probes. Notably, the CDs‐IR820 assembly, a carbonaceous material with high biocompatibility, successfully achieved the self‐reporting of PDT without the necessity of additional probes, thereby offering a novel paradigm for the design of self‐reporting materials.

## 3 CONCLUSION

In summary, we have successfully developed a smart light‐responsive theranostic system based on CDs (CDs‐IR820 assembly), offering outstanding PDT capability along with the real‐time monitoring. Notably, the CDs‐IR820 assembly effectively quenched the fluorescence and the PDT effect of CDs. Under the 750 nm laser irradiation, the CDs could be released and activated. Moreover, the released CDs not only produced ^1^O_2_ to eliminate tumor cells under xenon damp irradiation, but also functioned as a self‐reporter to monitor the PDT process. The light‐responsive self‐reporting theranostic platform based on CDs could achieve the most optimal treatment effects with minimal toxic side effects through the fine‐tuning of the illumination schedule, thereby presenting significant prospects for the precise PDT of tumor.

## CONFLICT OF INTEREST STATEMENT

The authors declare no conflicts of interest.

## ETHICS STATEMENT

There is no animal testing involved in this job, and thus no requirement for an ethics statement.

## Supporting information

Supporting Information S1

## Data Availability

The data that supports the findings of this study are available in the supplementary material of this article.
