# Supplementary material for: A NIR light activated self‐reporting carbon dots assembly as phototheranostics for tumor photodynamic therapy
Source: Smart Mol. 2024 Oct 21;3(2):e20240049. doi: 10.1002/smo.20240049 (PMC12262009; doi:10.1002/smo.20240049)
Supplement: Supplementary file 1 — Supporting Information S1 [file SMO2-3-e20240049-s001.docx]

Supporting Information

A NIR light activated self-reporting carbon dots assembly as phototheranostics for tumor photodynamic therapy

Ziyu Zhao, Tiejin Chen*, Jian Li, Xiaokuang Xue, Jiechao Ge*, Pengfei Wang

**Experimental Section**

**Chemical reagent**

New indocyanine green were purchased from J&K Scientific Ltd. Calcein AM, PI, Mito-Tracker Green, DAPI were obtained from Beijing Huali De Technology Co., Ltd. DCFH-DC was purchased from Shanghai Yare Biotech, Inc. All other chemical reagents and solvent were obtain from commercial source and used without further purification.

**Fabrication of carbon dots**

Firstly, polythiophene (PT2) was obtained in terms of our group reported synthetic protocols. Then 30 mg PT2 dispersed in 40 mL sodium hydroxide aqueous solution (20 mg/mL). The mixture was treated with ultrasonically around 30 min to get uniformly dispersed solution and then transferred into an autoclave, followed by heating to 170 °C for 24 h. After that, the reaction solution was cooled to room temperature. For purification, the reaction solution was dialyzed three times to remove sodium hydroxide solvent and unreacted PT2. Finally, the collected CDs were redispersed in deionized water and the concentration was around 0.5 mg/mL.

**Fabrication of CDs-IR820 assembly**

Firstly, the stock CDs solution was diluted to 0.02 mg/mL. And 5 mg of new indocyanine green was dispersed in 1 mL chloroform with ultrasonic wave. Then 200 μL new indocyanine green solution dropped into 5 mL CDs dilution. After that, the mixture solution was stirring at 40 °C, 300 rpm/min for 24 h to remove the organic solvent. The CDs-IR820 assembly were isolated through centrifugation at 16000 rpm/min and washing by pure water three times.

**Characterization of CDs-IR820 assembly**

Transmission electron microscopy (HT-7700, Hitachi) were used to visualize the CDs-IR820 assembly. UV-vis spectra and absorbance various were collected with a Hitachi U-3900 spectrophotometer. Fluorescence spectra and luminescence changes were obtained by a Hitachi

F-4600 fluorescence spectrophotometer. The hydrodynamic size and surface potential were measured through Malvern Zetasizer Nano ZEN3600.

**Detection of singlet oxygen generation**

In a typical chemical trapping method, ABDA was widely used as singlet oxygen trapping agent. Briefly, 60 μL ABDA Dimethyl sulfoxide solution (1 mg/mL) was added into 1.5 mL CDs-IR820 assembly aqueous solution. Then a 750 nm laser with the power density of 100 mW/cm^2^ and the xenon lamp were employed as the light source, respectively, to measure the absorbance decay of the mixture solution under different irradiation time. Besides, the deionized water and single IR820 irradiating by 750 nm laser were set as control group. Bruker E500 Electron Spin Resonance Spectroscopy was used to detect the generation of singlet oxygen as well. The aqueous solution of CDs-IR820 and the singlet oxygen trapping agent 2,2,6,6-tetramethylpiperidine-nitrogen (TEMP) were mixed in a specific ratio. In parallel, the changes of signal intensity under 750 nm laser or xenon lamp irradiation respectively were recorded.

**Cell culture**

The human-derived cervical cancer cells (HeLa) and mouse embryonic fibroblasts (3t3) were incubated in DMEM medium, which containing 10% FBS and double antibodies (100 μg/mL streptomycin and 100 μg/mL penicillin), at 37 °C in 5% CO_2_.

**Cellular uptake and controlled release**

For the cellular fluorescence imaging, HeLa cells were seeded in 35 mm cell culture dishes and co-incubated with CDs-IR820 assembly with 1 h, 1.5 h, 3 h and 4 h, respectively. Cellular luminescence imaging were captured using a Nikon C1si laser scanning confocal microscope at an excitation wavelength of 671 nm after being washed twice with PBS to eliminate non-specifically bound CDs-IR820. Then to further observe luminescence various after deassemblizing, using 750 nm laser with a power density of 100 mW/cm^2^ irradiated the HeLa cell with CDs-IR820 assembly.

**ROS detection in the cell**

HeLa cell were seeded into cell culture dishes and incubated with CDs-IR820 assembly with 3 h. Then 10 μL DCFH-DC probe (1.0 mM) was added into the HeLa cell and co-incubated for 30 min. After that, the HeLa cell was irradiated under 750 nm laser with 3 min, followed by xenon lamp irradiation. The whole process was recorded by Nikon C1si laser scanning confocal microscope (Ex = 488 nm, Em = 500-530 nm).

**Evaluation of antitumor ability**

Firstly, HeLa and 3t3 cell was seeded in a 96-well plate and incubated in 5% CO_2_ at 37 °C overnight. Then the HeLa and 3t3 cells co-incubated with different concentration of CDs- IR820 assembly from 0 to 200 μg/mL, respectively. After another 24 h, the fresh medium with 20 μL of 3-(4,5-dimethylthiazol-2-yl)-2,5-diphenyltetrazolium bromide (MTT, 5 mg/mL in PBS) was used to replace the old medium. The plate was then incubated for an additional 4 hours. Finally, the medium change to DMSO and the absorbance of each cell at 490 nm was measured using microplate reader.

For the PDT, excepting irradiating with 750 nm laser (100 mW/cm^2^) or xenon lamp for 15 min before the standard MTT assay, the rest of experimental procedures were same as above. To further confirm the efficiency of PDT, calcein AM/PI staining was also carried out. Similar with the drug addition process of MTT assays, after incubation 24 h, 1 μL calcein (1 mM) and 5 μL PI (1 mM) were added into the cell. Following another 20 min incubation, Nikon C1si laser scanning confocal microscope was used for imaging.

**Figure S1.** The size distribution histogram of CDs-IR820 assembly derived from TEM image.


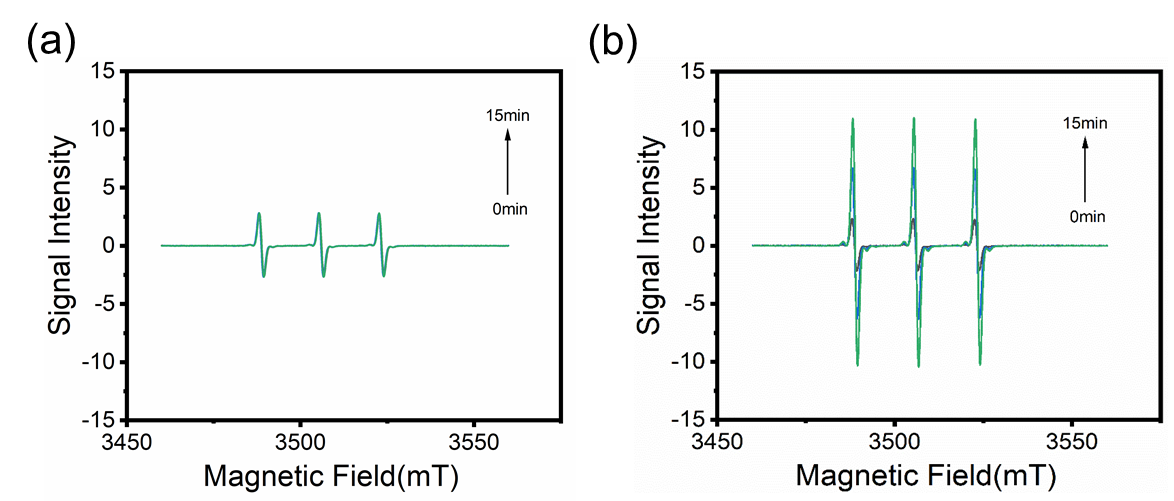


**Figure S2.** The spectra of ESR signal from CDs-IR820 assembly (a) under 750 nm laser irradiation; (b) under 750 nm laser followed with the white light irradiation.


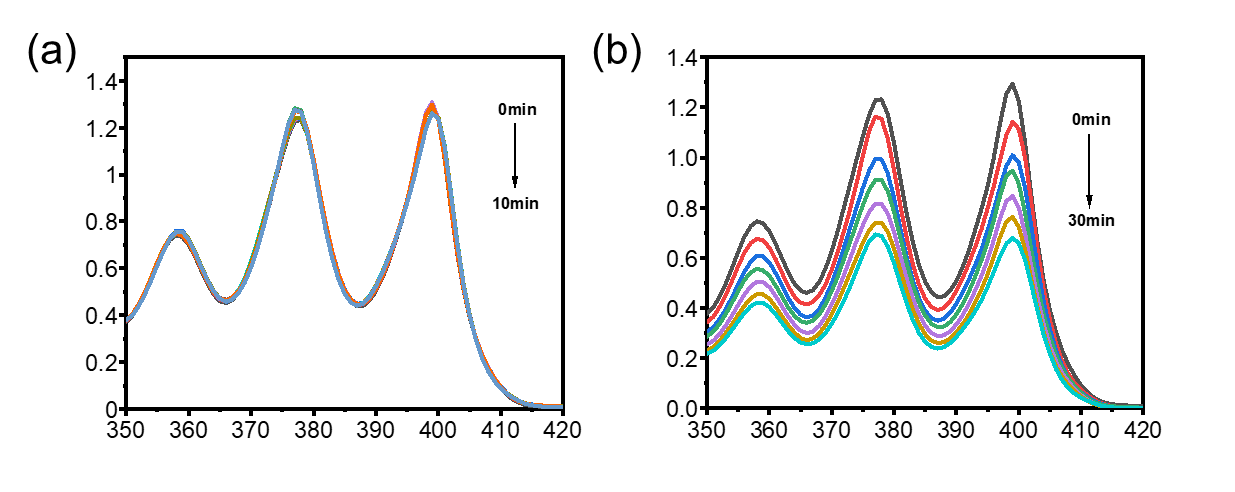


**Figure S3.** Absorption spectra of ABDA mixed with CDs-IR820 assembly (a) under 750 nm laser irradiation; (b) under 750 nm laser followed with the white light irradiation.


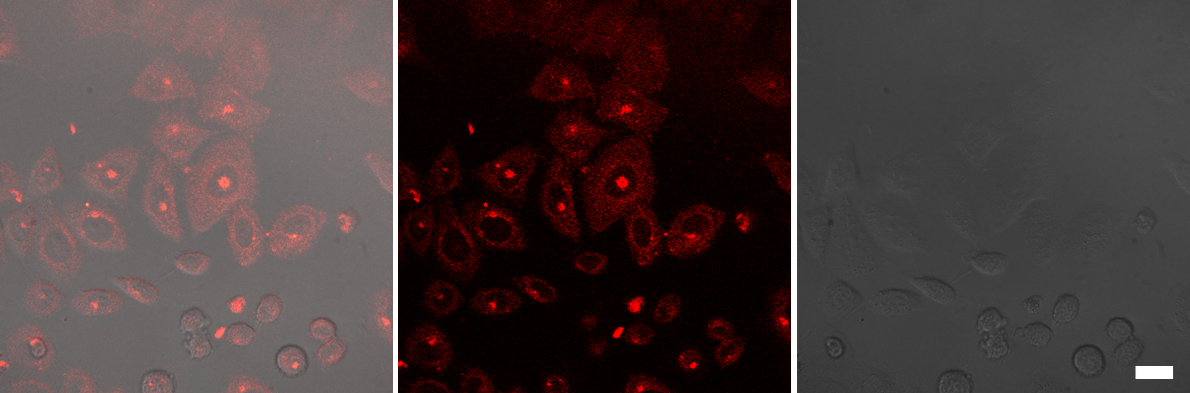


**Figure S4.** Confocal microscopy images of HeLa cells incubated with CDs-IR820 assembly with prolonged observation time. Scale bar: 20 μm.


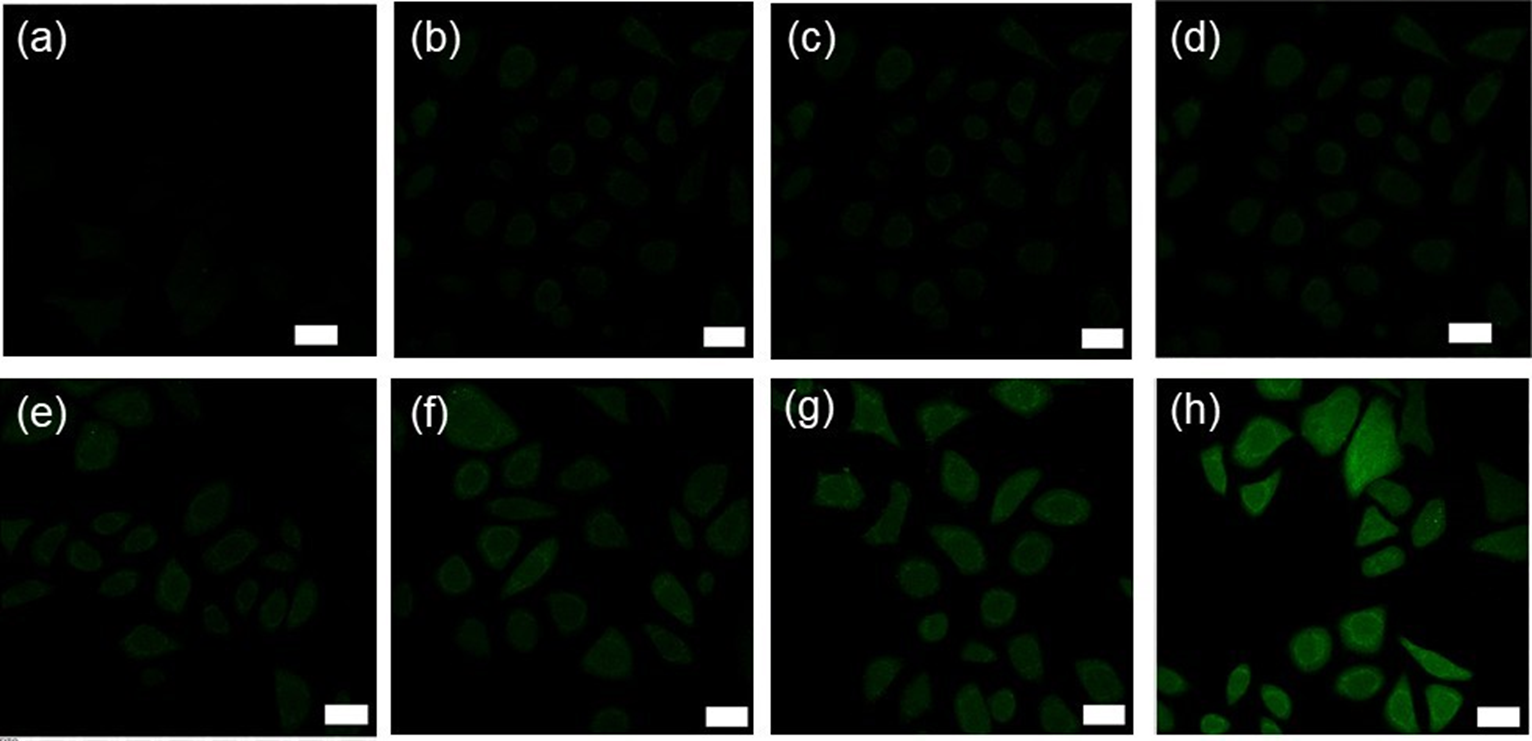


**Figure S5.** Time-dependent images of reactive oxygen species production detected by DCFH-DA in HeLa cells: (a)-(d) under irradiation with 750 nm laser for different time (0, 1, 2 and 3 min) and (e)-(h) under irradiation with 750 nm laser (3 min) followed with the white light for different time (0.5, 1, 1.5 and 3 min). Scale bar: 20 μm.

*
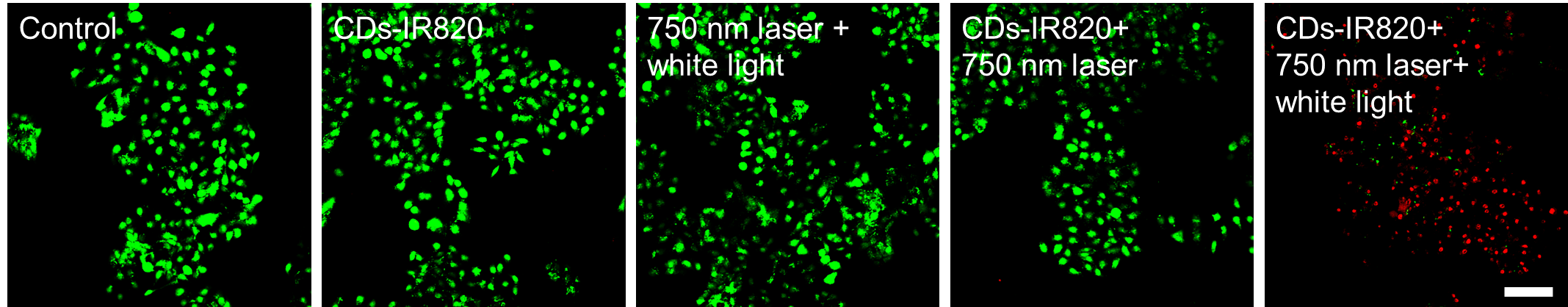
*

**Figure S6.** The FL image of Calcein AM/PI co-staining HeLa cells under different treatments. Scar bar: 100 μm.

**Table S1.** The summary of the recent studies on self-reporting PDT agents.

| Materials categories | Samples | Mechanism of self-reporting | References |
| --- | --- | --- | --- |
| Organic small molecules | TPE-4EP+  (AIE molecule) | Mitochondria-to-nucleus translocation | ^[1]^ |
|  | MTPA-SPP  (Incorporate a PS into a DNA-specific dye SPP) | Mitochondrion-to-nucleus migration | ^[2]^ |
|  | Ru4  (Ru(II) polypyridyl complexes) | Lysosomes-to-mitochondria  translocation | ^[3]^ |
|  | TAB-6-amyl  (Triarylboron-based fluorescent probes) | Strong fluorescence in apoptotic cells instead of no fluorescence for living cells | ^[4]^ |
|  | NG-cRGD  (N2O-type benzopyrromethene boron complexes) | Conversion of NIR-to-red fluorescence | ^[5]^ |
| Carbon-based materials | CDs | The migration between mitochondrion and nucleus (only monitor the cell viability without PDT) | ^[6]^ |
| Black  phosphorus | TPBP  (Black phosphorus loaded a two-photon fluorescent nanoprobe) | ^1^O_2_ stimulated a two-photon molecule to emit fluorescence signals for feedback of ^1^O_2_ generation | ^[7]^ |
| COF | UCCOFs-1  (A ROS indicator was loaded on UCCOFs) | Emit singlet oxygen-correlated luminescence | ^[8]^ |
| Gold nanoparticles | AuNP nanoprobes  (AuNPs loaded NCL fluorescent probe) | “Light-on” NCL fluorescent nanoprobe to track the dynamic distribution and expression of NCL | ^[9]^ |

References

[1] T. Zhang, Y. Li, Z. Zheng, R. Ye, Y. Zhang, R. T. K. Kwok, J. W. Y. Lam, B. Z. Tang, *J. Am. Chem. Soc.* **2019**, 141, 5612.

[2] T. Xia, Z. Xia, P. Tang, J. Fan, X. Peng, *J. Am. Chem. Soc.* **2024**, 146, 12941-12949.

[3] K. Qiu, Y. Wen, C. Ouyang, X. Liao, C. Liu, T. W. Rees, Q. Zhang, L. Ji, H. Chao, *Chem. Commun.* **2019**, 55, 11235.

[4] J. Liu, H. Chen, B. Wang, Y. Luo, G. Yang, S. Zhang, S. Li, *Anal. Chem.* **2022**, 94, 8483.

[5] C. Wang, Y. Sun, S. Huang, Z. Wei, J. Tan, C. Wu, Q. Chen, X. Zhang, *J. Am. Chem. Soc.* **2023**, 145, 13099-13113.

[6] S. Guo, Y. Sun, J. Li, X. Geng, R. Yang, X. Zhang, L. Qu, Z. Li, *ACS Appl. Bio Mater.* **2021**, 4, 928.

[7] K. Guan, P. Wang, F. Zhou, Y. Wang, H. W. Liu, Q. Xie, G. Song, X. Yin, S. Huan, X. B. Zhang, *Chem. Commun.* **2020**, 56, 14007.

[8] P. Wang, F. Zhou, K. Guan, Y. Wang, X. Fu, Y. Yang, X. Yin, G. Song, X. B. Zhang, W. Tan, *Chem. Sci.* **2020**, 11, 1299.

[9] J. Kong, X. Ju, G. Qi, J. Wang, X. Diao, B. Wang, C. Zhang, J. Li, Y. Jin, *Anal. Chem.* **2024**, 96, 926.
